# Editorial: Impacts of public-private collaborative research on Alzheimer's disease: the case of the innovative medicines initiative

**DOI:** 10.3389/fneur.2023.1321225

**Published:** 2023-11-29

**Authors:** Elisabetta Vaudano, Jean Georges, Martin Hofmann-Apitius, Donald Lo

**Affiliations:** ^1^The Innovative Health Initiative Joint Undertaking, Brussels, Belgium; ^2^Alzheimer Europe, Luxembourg, Luxembourg; ^3^Department of Bioinformatics, Fraunhofer Institute for Algorithms and Scientific Computing (SCAI), Bonn, Germany; ^4^EATRIS, Amsterdam, Netherlands

**Keywords:** partnership, patient, networks, dementia, data sharing, Alzheimer's, collaboration

The number of people living with dementia, mostly due to Alzheimer's disease (AD), has been recently estimated at more than 50 million globally (1), and this is only expected to rise further and substantially as the world's population ages. If we look at the entire AD continuum, global prevalence estimates increase to >400 million people, or ~22% of all people aged 50+ (2). While these statistics are staggering, most of those affected are in the earliest stages of the disease, and this suggests that there remains a real window of opportunity for tackling the AD challenge and introducing prevention and risk reduction strategies.

For people with mild cognitive impairment from AD and early AD dementia, the first disease-modifying therapies have been approved by the Food & Drug Administration (FDA) and in Japan (3, 4). However, only a fraction of patients will benefit from these first therapies due to the biological heterogeneity of the disease and the lack of readiness of healthcare systems. In addition, the modest effectiveness of these new treatments will need to be balanced against their side effects and the burden of current dosing regimens (e.g., infusions every 2–4 weeks) (5).

Despite these positive developments, much more needs to be done to de-risk the dementia area and transform scientific knowledge into concrete patient outcomes. “Silos” must be broken and communities set to work with a common agenda, across the public-private space, to co-create solutions. This will avoid a waste of resources on both the public and industry sides and foster collaboration instead of fragmentation of efforts. This approach of “radical collaboration” has been very successfully implemented by the public-private partnerships (PPPs) of the European Innovative Medicines Initiative (IMI) and its successor, the Innovative Health Initiative (IHI) (6).

This Research Topic volume provides a flavor of the achievements and learnings gained through the PPP projects in the IMI/IHI AD portfolio (7). The 11 papers cover a broad range of challenges and opportunities in AD, focusing on those critical for the development of new diagnostic approaches and treatments.

Difficulty in finding and accessing high-quality data and samples is hindering the validation of biomarkers for use in developing both diagnostics and treatments in AD. The EPND (Bose et al.) (8) collaborative platform uniquely enables the sharing, reuse, and large-scale analysis of the high-quality data and samples needed to accelerate biomarker validation, while maintaining robust protection for data subjects and giving data and research additional usability, beyond original studies.

While new biomarkers are emerging, amyloid detection with positron emission tomography (PET) remains the workhorse for characterizing the start and spread of AD in both trials and clinical practice. The AMYPAD project (9) significantly furthered our understanding of amyloid deposition in the brain and the optimal methodology to measure this process across tracers, highlighting the utility of amyloid PET for both initial diagnosis and prognosis, and enabling optimal therapy monitoring and/or patient management (Collij et al.) .

In clinical trials for AD and other neurodegenerative diseases, there is a critical need for novel outcome measurements of sufficient sensitivity, especially in the early stages of disease and for studying disease progression. In this context, digital endpoints could be game changers. Brem et al. share important learnings and findings from the RADAR-AD (10), MOBILIZE-D (11), and IDEA-FAST (12) projects on the value of remote technologies for assessing neurodegenerative diseases. They discuss the feasibility, acceptability, and usability of digital assessments, as well as the challenges, and regulatory learnings, emphasizing the importance of public/patient involvement and inter-project exchange as well as data and algorithm sharing.

Innovation in clinical trial design is a must for speeding up treatment development for AD, Saunders et al., and EPAD (13) pioneered the concept of platform trials, delivering key learnings and open assets. These include the EPAD longitudinal cohort study (LCS) data and biobank, and the trial network is now incorporated into the Global Alzheimer's Platform (GAP) for a truly global impact.

Fragmentation of data, results, and initiatives remains a major underlying issue in the AD field (and beyond) and is one of the key factors slowing down progress. The EHDEN (14) project is spearheading a new approach for the aggregated analyses of hundreds of millions of electronic healthcare records (EHRs) with speed, transparency, and privacy protection that can represent a true paradigm shift in the conduct of observational studies across many disease areas. Díaz et al. NEURONET (15) coordinated, harmonized, and integrated data and results from IMI AD projects, delivering important assets for the research community, like the knowledge base (16). Additionally, the NEURONET data-sharing Working Group (Bradshaw et al.) provided valuable examples of good practices and recommendations on how to overcome obstacles to data sharing, from organizational and technical issues to socio-technical hurdles. Provocatively, they consider whether we should think about data collaboration, rather than only data sharing.

Recent advances in diagnostics and the approval of new pharmaceutical treatments for AD herald the beginning of precision medicine in the AD field. Progress in implementing biomarkers, clinical trial design, and endpoints and in data sharing will further increase the offer of treatments. However, their implementation will challenge already over-burdened healthcare systems. The IHI project PROMINENT (17) is developing a digital platform and clinical support system that integrates diverse data and real-world evidence across each aspect of the care pathway, from diagnosis to treatment, for guiding treatment and assessing its benefits (Tate et al.). Uniquely, this will be achieved in a truly collaborative effort of dementia researchers, medical professionals, dementia patients, and their care partners with the developers of innovative health technologies.

PPPs funded under IMI represent ideal and powerful vehicles to integrate research efforts across the public and private sectors in the AD space, creating knowledge of high transferability, as demonstrated by the AETIONOMY outputs (18, 19) that have been further developed not only for AD (20) but also in the COVID-PHARMACOME (21). Analyzing the collaborative networks across IMI's AD projects, O'Rourke et al. and Hawksworth et al. highlight their impacts, suggest areas requiring improvement, and offer recommendations for future PPPs. Meanwhile, North et al. document how the IMI model has boosted multi-disciplinary collaboration across Europe (within industry, within academia, and across industry-academia) like never before. North et al. also note that the express goal of each project is to meet the needs of patients.

Public Involvement (PI), i.e., the active involvement of people with dementia in dementia research projects other than as research participants, is a key for advancing the AD field. Georges et al. highlight the critical role of patients as research partners, emphasizing current gaps and highlighting successful PI examples from IMI projects.

We hope that you will enjoy reading this Research Topic issue and will benefit from its messages and learnings.

## Author contributions

EV: Conceptualization, Writing – original draft, Writing – review & editing. JG: Writing – review & editing. MH-A: Writing – review & editing. DL: Writing – review & editing.
